# The Neuroprotective Action of Amidated-Kyotorphin on Amyloid β Peptide-Induced Alzheimer’s Disease Pathophysiology

**DOI:** 10.3389/fphar.2020.00985

**Published:** 2020-07-09

**Authors:** Rita F. Belo, Margarida L. F. Martins, Liana Shvachiy, Tiago Costa-Coelho, Carolina de Almeida-Borlido, João Fonseca-Gomes, Vera Neves, Hugo Vicente Miranda, Tiago F. Outeiro, Joana E. Coelho, Sara Xapelli, Cláudia A. Valente, Montserrat Heras, Eduard Bardaji, Miguel A. R. B. Castanho, Maria José Diógenes, Ana M. Sebastião

**Affiliations:** ^1^ Instituto de Farmacologia e Neurociências, Faculdade de Medicina, Universidade de Lisboa, Lisbon, Portugal; ^2^ Instituto de Medicina Molecular João Lobo Antunes, Faculdade de Medicina, Universidade de Lisboa, Lisbon, Portugal; ^3^ Cardiovascular Autonomic Function Lab, Centro Cardiovascular da Universidade de Lisboa, Lisbon, Portugal; ^4^ Instituto de Bioquímica, Faculdade de Medicina, Universidade de Lisboa, Lisbon, Portugal; ^5^ CEDOC, Chronic Diseases Research Center, NOVA Medical School, Faculdade de Ciências Médicas, Universidade Nova de Lisboa, Lisboa, Portugal; ^6^ Department of Experimental Neurodegeneration, Center for Biostructural Imaging of Neurodegeneration, University Medical Center Göttingen, Göttingen, Germany; ^7^ Max Planck Institute for Experimental Medicine, Göttingen, Germany; ^8^ Translational and Clinical Research Institute, Faculty of Medical Sciences, Newcastle University, Framlington Place, Newcastle Upon Tyne, United Kingdom; ^9^ Laboratori d'Innovació en Processos i Productes de Síntesi Orgànica (LIPPSO), Departament de Química, Universitat de Girona, Girona, Spain

**Keywords:** amidated-kyotorphin, Alzheimer’s disease, amyloid β peptide, novel object recognition test, Y-Maze alternation test, long-term potentiation, memory, synaptic plasticity

## Abstract

Kyotorphin (KTP, l-tyrosyl-l-arginine) is an endogenous dipeptide initially described to have analgesic properties. Recently, KTP was suggested to be an endogenous neuroprotective agent, namely for Alzheimer’s disease (AD). In fact, KTP levels were shown to be decreased in the cerebrospinal fluid of patients with AD, and recent data showed that intracerebroventricular (i.c.v.) injection of KTP ameliorates memory impairments in a sporadic rat model of AD. However, this administration route is far from being a suitable therapeutic strategy. Here, we evaluated if the blood-brain permeant KTP-derivative, KTP-NH_2_, when systemically administered, would be effective in preventing memory deficits in a sporadic AD animal model and if so, which would be the synaptic correlates of that action. The sporadic AD model was induced in male Wistar rats through i.c.v. injection of amyloid β peptide (Aβ). Animals were treated for 20 days with KTP-NH_2_ (32.3 mg/kg, intraperitoneally (i.p.), starting at day 3 after Aβ administration) before memory testing (Novel object recognition (NOR) and Y-maze (YM) tests). Animals were then sacrificed, and markers for gliosis were assessed by immunohistochemistry and Western blot analysis. Synaptic correlates were assessed by evaluating theta-burst induced long term potentiation (LTP) of field excitatory synaptic potentials (fEPSPs) recorded from hippocampal slices and cortical spine density analysis. In the absence of KTP-NH_2_ treatment, Aβ-injected rats had clear memory deficits, as assessed through NOR or YM tests. Importantly, these memory deficits were absent in Aβ-injected rats that had been treated with KTP-NH_2_, which scored in memory tests as control (sham i.c.v. injected) rats. No signs of gliosis could be detected at the end of the treatment in any group of animals. LTP magnitude was significantly impaired in hippocampal slices that had been incubated with Aβ oligomers (200 nM) in the absence of KTP-NH_2_. Co-incubation with KTP-NH_2_ (50 nM) rescued LTP toward control values. Similarly, Aβ caused a significant decrease in spine density in cortical neuronal cultures, and this was prevented by co-incubation with KTP-NH_2_ (50 nM). In conclusion, the present data demonstrate that i.p. KTP-NH_2_ treatment counteracts Aβ-induced memory impairments in an AD sporadic model, possibly through the rescuing of synaptic plasticity mechanisms.

## Introduction

Kyotorphin (KTP) is an endogenous dipeptide composed by tyrosine and arginine (l-tyrosyl-l-arginine) residues, first described as a powerful analgesic molecule (Takagi et al., 1979a; Takagi et al., 1979b). Given its analgesic properties, KTP has been investigated as a drug for pain treatment (Ribeiro et al., 2011b; Santos et al., 2013). Although mechanisms underpinning KTP-induced analgesic effects are still not entirely understood, some authors argue that KTP binds to a specific Gi-coupled protein receptor (KTPr), which despite numerous efforts, has never been isolated (Ueda et al., 1989). Nevertheless, it is well-known that KTP triggers the release of met-enkephalins (met-enk) and β-endorphins in a naloxone-reversible opioid receptors mediated-mechanism (Takagi et al., 1979a; Shiomi et al., 1981; Ueda et al., 1982; Oliveira et al., 2016), an effect antagonized by the dipeptide l-Leu-l-Arg (a KTPr antagonist) (Ueda et al., 1989; Kawabata et al., 1992).

Interestingly, despite the high expression of KTP in the cortex, the levels of enkephalins and of opioid receptors are low, suggesting other non-opioid physiological actions for KTP (Ueda et al., 1980). Remarkably, KTP has been pointed as a possible neuroprotective factor (Dzambazova and Bocheva, 2010), emerging as a novel drug to be explored for Alzheimer´s disease (AD) and other therapeutic applications.

AD is a chronic progressive neurodegenerative disease and the most common cause of dementia in elderly. The presence of senile plaques (amyloid beta (Aβ) peptide) and neurofibrillary tangles (hyperphosphorylated tau (p-Tau) protein) in the brain are the molecular hallmarks of the disease (Price et al., 1991; Citron, 2010). AD represents a challenge for drug discovery since effective neuroprotective treatments are still needed. It was reported that AD patients present decreased levels of KTP in cerebrospinal fluid (CSF) (Santos et al., 2013) suggesting the increase in KTP levels as a possible therapeutic strategy.

Despite the encouraging recent data showing that intracerebroventricular (i.c.v) injection of KTP ameliorates memory impairments in a sporadic AD rat model (Angelova et al., 2019), its weak activity when administered systemically (Chen et al., 1998) renders KTP as an unrealistic pharmacological tool to fight AD. Amidated-kyotorphin (KTP-NH_2_), is a KTP derivative capable of crossing the blood brain barrier (BBB) (Ribeiro et al., 2011a; Ribeiro et al., 2011b), and with efficacy to decrease neuronal damage induced by cerebral hypoperfusion (Sá Santos et al., 2016). Altogether, these findings prompted us to investigate whether systemic administration of KTP-NH_2_ would be effective to ameliorate memory impairment in an animal model of sporadic AD, and if so, which are the synaptic mechanisms operated by KTP-NH_2_ to protect synapses against Aβ-induced toxicity.

## Materials and Methods

### Drugs

#### Amyloid β Peptide

For *in vivo* experiments, Amyloid β (Aβ) peptide 1 to 42 (Aβ_1–42_) (H-1368, Bachem Bubendorf, Switzerland) was dispersed in water at a concentration of 2.25 mg/ml.

In order to prepare oligomeric species of Aβ_1–42_ (Aβ_olig_), Aβ_1–42_ (1 mg/ml) (A-42-T, GenicBio, Shanghai, China) was suspended in phosphate-buffered saline (PBS), supplemented with 0.025% ammonia solution and adjusted to a final pH 7.2 (HCl). Species separation was based on an ultrafiltration process, as previously described (Giuffrida et al., 2009). Briefly, Aβ_1–42_ (220 µM) was allowed to oligomerize by constant shaking at 600 rpm, at 37°C for 16 h and ultracentrifuged (40,000*g*, 30 min) for separation of fibrils (pellet). The supernatant was further separated in centrifugal filters (30 kDa Amicon Ultra). The concentration of the retained fraction, corresponding to oligomers > 30 kDa, was spectrophotometrically determined (ϵ_280_ = 1490 M^−1^ cm^−1^). Oligomers aliquots (120–220 µM) were immediately stored at −80°C until further use.

In addition, *in vitro* experiments using primary neuronal cultures were performed using the Aβ fragment 25–35 (Aβ_25–35_) (Bachem, Bubendorf, Switzerland). Aβ_25–35_ represents the biologically active region of Aβ and induces the same molecular and cellular dysfunction as Aβ_1–42_ species, being this effect similar to what has been observed in AD brains (Pike et al., 1995; Kaminsky et al., 2010). Stock solutions of Aβ_25–35_ were prepared in MilliQ water to a final concentration of 1 mg/ml.

#### KTP-NH_2_ Peptide

KTP-NH_2_ peptide was synthesized as previously described (Ribeiro et al., 2011b). For *in vivo* experiments, KTP-NH_2_ was dissolved in physiological saline solution (0.9% NaCl, vehicle solution), as a 100 mM stock solution, and it was administered at a dose of 32.3 mg/kg, at a volume of 1 ml/kg. The selected dose was based on previous results regarding KTP-NH_2_ analgesic action profile (Ribeiro et al., 2011b; Ribeiro et al., 2013). For *ex vivo* and *in vitro* experiments, KTP-NH_2_ was prepared in previously filtered and sterile milliQ water as 1 and 5 mM stock solutions, respectively.

### Intracerebroventricular Injection of Aβ Peptide

Male Wistar rats (8–10 weeks), purchased from Charles River Laboratories (Lyon, France), were housed in a group of 2 per cage and maintained under controlled conditions (20 ± 2°C; 14:10 h light/dark cycle, lights on between 7:00 am and 9:00 pm). All animals had unrestricted access to food and water. The handling of animals and all described procedures were conducted according to the European Community (86/609/EEC; 2010/63/EU; 2012/707/EU) and Portuguese (DL 113/2013) legislation for the protection of animals used for scientific purposes, and they were approved by the Ethical Committee for Animal Research of Instituto de Medicina Molecular João Lobo Antunes (iMM), Faculty of Medicine, University of Lisbon, and the Portuguese Competent Authority for Animal Welfare (DGAV) in Portugal.

The animal model of AD was created based on the Aβ_1–42_ i.c.v. injection method, as previously described (Canas et al., 2009; Zhang et al., 2015). Surgical procedures were performed when animals reached 230 to 320 g and during the light period. Briefly, animals were anesthetized with isoflurane (2–3% in O_2_) using a RC2 Rodent Anesthesia System (VetEquip Inc., California, USA), firstly using a plexiglas chamber and thereafter maintained *via* facial mask. EMLA^®^ cream was applied in the ear canal, and Bupivacaine Hydrochloride 0.25% (8 mg/kg, SC) was administered at the incision site for local anesthetics. Lacryvisc^®^ (Carbomer 974P 0.3%) was applied on the eyes to avoid dehydration. Buprenorphine (0.05 mg/kg, SC) was also administered pre-emptively for general analgesia, so it would be already in action when animals recovered from anesthesia.

**Figure 1:**
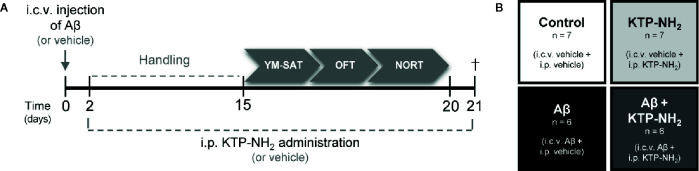
Experimental design. **(A)** Animals were i.c.v. injected with Aβ (Aβ_1–42_) or vehicle (water) at day 0. On day 2, KTP-NH_2_ or vehicle (saline solution) i.p. administration began, simultaneously with handling. Behavioral tests were performed between days 15 and 20: Y-Maze Spontaneous Alternation test (YM-SAT), Open Field test (OFT), and Novel Object Recognition test (NORT). On day 21 animals were sacrificed (†) for molecular analyses (WB and IHC). **(B)** Four experimental groups were tested: Control (i.c.v. and i.p. vehicle administration, n = 7); Aβ (i.c.v. Aβ administration and i.p. vehicle treatment, n = 6); KTP-NH_2_ (i.c.v. vehicle administration and i.p. KTP-NH_2_ treatment, n = 7), and Aβ + KTP-NH_2_ (i.c.v. Aβ administration and i.p. KTP-NH_2_ treatment, n = 6).

Twelve animals were i.c.v. injected with Aβ peptide (2.25 mg/ml) in 5 µl and 14 were injected with 5 µl of water (vehicle) at day zero. Injections were performed with a 33-gauge Hamilton microsyringe (Hamilton Company, Nevada, USA) using a microinjection pump (World Precision Instruments, Inc., Florida, USA) with a rate of 500 nl/min, in the right lateral ventricle using a stereotactic system (anteroposterior, −0.84 mm from bregma; medial/lateral, 1.5 mm; and dorsal/ventral, −3.5 mm). Injections lasted 10 min, and the needle with the syringe was left in place for 2 min after the injection to ensure complete infusion of Aβ. Animal body temperature was kept constant at 37°C using a heating pad. A timeline of all the experimental events is depicted in **Figure 1A**. Previous work reported soluble forms of Aβ on the hippocampus of i.c.v. Aβ injected animals (Canas et al., 2009), thus showing Aβ diffusion through this brain region. Compared with transgenic models of AD, the i.c.v. Aβ-induced sporadic model of AD is more relevant for the study of Aβ-induced pathophysiological traits of AD. Moreover, the transgenic models of AD are usually used to study the familial form of the disease, which barely represents 5% of AD cases (Lecanu and Papadopoulos, 2013).

### Chronic Intraperitoneal KTP-NH_2_ Treatment

Thirteen animals underwent a chronic 18-day treatment regimen of KTP-NH_2_ (32.3 mg/kg, single i.p. dose/day), starting the second day after i.c.v. administration of Aβ_1–42_ (Aβ + KTP-NH_2_ group, n=6) or vehicle (KTP-NH_2_ group, n=7), and lasting until sacrifice. The remaining thirteen animals received the vehicle solution following the same treatment regimen: Aβ + vehicle (Aβ group, n = 6) and vehicle + vehicle (control group, n = 7). The experimental groups of animals are depicted in **Figure 1B**. During the last 5 days of i.p. treatments (KTP-NH_2_ or vehicle), animals were tested in the behavioral paradigms identified below. During behavioral assessment days, injections were performed after animal testing and before return to the animal house.

### Behavioral Testing

Behavioral tests were performed from the fifteenth day after Aβ_1–42_ injection, following a previously described protocol (Cunha et al., 2008; Canas et al., 2009). The handling period coincided with the first 13 days of KTP-NH_2_ treatment, where animals were handled for a few minutes before the i.p. injection so that they became used to the experimenter and to the testing room. All behavioral tests were carried out between 9:00 am and 6:00 pm. All the apparatus used were cleaned with 70% ethanol between animals switching. After placing the animal inside the behavioral apparatus, the experimenter immediately left the room. The experimenter conducting behavioral analysis was blinded to treatment conditions.

Tests were performed in the following order: Y-Maze Spontaneous Alternation Test (YM-SAT), Open Field Test (OFT), and Novel Object Recognition Test (NORT) (**Figure 1A**). The YM-SAT was performed before the NORT to evaluate working memory for a less complex paradigm first. Since OFT and NORT use the same testing arena, the OFT was performed during the first 5 min of the first day of NORT habituation period (first contact with the arena).

#### Y-Maze Spontaneous Alternation Test (YM-SAT)

The spontaneous alternation version of the Y-Maze test evaluates spatial working memory by taking advantage of the willingness of rodents to explore new environments. The testing protocol for YM-SAT has been described in detail previously (Maurice et al., 1996). The wood-made maze used was composed of 3 arms (30 x 10 x 20 cm each), converging to an equal angle. Visual cues were placed on the walls of the maze. Briefly, without prior habituation, animals were individually placed at the end of one arm and allowed to freely move through the maze for 8 min. Two independent investigators visually recorded arm entries, and the comparison between recordings showed total agreement.

An entry was considered valid when all four limbs of the animal were within the arm. An alternation was defined as entries in all three arms on consecutive occasions. The number of maximum possible alternations for each animal was therefore the total number of arm entries minus two. The percentage of spontaneous alternation was calculated as actual alternations/maximum alternations ×100. In addition, the total number of arm entries was used as a measure of locomotor activity.

#### Open Field Test (OFT)

The OFT is widely used to assess individual spontaneous locomotor activity and anxiety-like behavior when animals are introduced to a novel environment (Prut and Belzung, 2003; Bailey and Crawley, 2009; Castanheira et al., 2018). Importantly, this test allows to detect bias in animal behaviour that could affect performance on NORT, since locomotor activity can impact exploratory drive (Broadhurst, 1957; Broadhurst, 1958). The open field arena consisted of an empty square wood box (67 x 67 x 51 cm height), virtually divided into three concentric squares: a peripheral, an intermediate and a central zone. The testing protocol has been described in detail previously (Gould et al., 2009). Briefly, animals were individually placed in the center of the arena and allowed to freely move for 5 min. The behaviour was video-recorded and analyzed by the tracking software Smart^®^ (version 2.5; Panlab, Barcelona, Spain). The reference point used for the animal tracking position was defined as the center of the animal dorsum. Both total travelled distance (cm) and average velocity (cm/s) were measured to assess locomotor activity. In addition, the percentage of time spent in the arena periphery was used as an indicator of the level of anxiety-like behavior. All animals were tested only once.

#### Novel Object Recognition Test (NORT)

Considering that episodic long-term memory is the predominant cognitive deficit in AD, animals were subjected to the NORT (Antunes and Biala, 2012). To assess long-term memory a retention interval (RI) of 24 h was used. This test consists of three phases: habituation, familiarization (training), and test. The habituation phase consisted of 3 sessions of habituation to the arena (15 min each day). During both familiarization and test phases, two objects were added to the same arena, always in the same place. The objects used in this test were translucent glass bottles and brown glass bottles without labels, that were similar in size and shape, but which the animals were able to discriminate between them. To prevent coercion to explore the objects, animals were individually placed in the middle of the opposite wall where objects were, with their backs to them, and allowed to freely move for 5 min. In the familiarization phase, animals were presented with two equal objects, which we herein name as ‘familiar’ objects. During the familiarization phase, chosen objects were counterbalanced between animals within the same experimental group to reduce any object preference effect. Similarly, object localization was counterbalanced between both arena sides, to eliminate possible preference confounds for a specific side of the arena. Following sample-objects exposure, animals returned to the home cage for 24 h. In the test phase, one of the previously experienced objects (now considered familiar objects) was substituted by a new object (considered the novel object). Exploration was scored when the animal touched an object with its forepaws or snout, bit, licked, or sniffed the object from a distance of no more than 1.5 cm. Running around the object or climbing on it was not recorded as exploration. Animal movements were recorded using the SMART^®^ video-tracking software (version 2.5; Panlab, Barcelona, Spain). A post-analysis was conducted to refine the results obtained by software measures. Two independent investigators made the post-analysis with a high degree of concordance between them.

Three indexes were calculated in order to evaluate both preference between objects and recognition of the novel object: 1) the object preference index, which is the ratio between the time spent exploring one object over the total time spent exploring both objects [object 1 or object 2/(object 1 + object 2)], and it is assessed both in the training and test phases; 2) the object recognition index, which is the ratio between the time spent exploring the novel object and the total time spent exploring both objects [novel/(novel + familiar)], and it is an index of retention, used for analysis of the test phase performance; and finally, 3) the object discrimination index, which is the ratio between the difference between the time spent exploring the novel and the familiar object, and the total time spent exploring both objects [(novel − familiar)/(novel + familiar)], allowing an easier visualization of data since no memory retention scores as zero (Antunes and Biala, 2012).

### Western Blot

After behavioral tests, three/four animals from each group were deeply anesthetized with isoflurane (Esteve, Barcelona, Spain) and decapitated. Their brains were quickly removed and placed in ice-cold aCSF, continuously oxygenated (O_2_/CO_2_: 95%/5%) to isolate both hippocampi. The left and the right hippocampi were individually frozen in liquid nitrogen and stored at −80°C. The two hippocampi were analyzed separately to allow the separate quantification of the hippocampus of the side of the i.c.v. injection (right, ipsilateral) and of the contralateral to it. Since both hippocampi could be differently exposed to Aβ, they could display different degrees of gliosis. Using a Potter homogenizer, tissue homogenates were prepared from frozen samples by solubilizing them in Radio-Immunoprecipitation Assay (RIPA) buffer: 50 mM Tris-base (pH 7.5), 150 mM NaCl, 5 mM Ethylenediaminetetraacetic Acid (EDTA), 0.1% *Sodium Dodecyl Sulphate* (SDS), and 1% Triton X-100, supplemented with phosphatase inhibitors: 10 nM NaF; 5 mM Na_3_VO_4_ and a protease inhibitor cocktail (Complete Mini-EDTA free from Roche, Penzberg, Germany). Samples were centrifuged at 13,000*g*, 4°C for 10 min, and the supernatant was collected and placed in fresh tubes. Protein concentration was quantified through Bradford method, using Bio-Rad DC reagent (Bio-Rad Laboratories, Berkeley, CA, United States). All samples were prepared with the same amount of total protein (35 µg), by adding a loading buffer (350 mM Tris-HCl (pH 6.8), 10% SDS, 30% glycerol, 600 mM Dithiothreitol, 0.06% bromophenol blue), and then boiled at 95°C for 5 min. Next, samples and the molecular weight marker (PageRuler™ Plus Prestained Protein Ladder, 10 to 250 kDa, ThermoFisher Scientific, Massachusetts, USA) were loaded and separated on 12% SDS–polyacrylamide gel electrophoresis (SDS–PAGE) within a standard migration buffer (25 mM Tris-base (pH 8.3), 192 mM Glycine, 10% SDS), at a constant voltage between 80 and 120 mV. Subsequently, proteins were electrotransferred, at 400 mA for 1 h 30 min, from the gel to a polyvinylidene difluoride (PVDF) membrane (GE Healthcare, Buckinghamshire, United Kingdom), previously activated with methanol for 5 min, within the standard buffer (25 mM Tris (pH 8.3), 192 mM Glycine, 15% methanol) for wet transfer conditions. Afterward, membranes were stained with Ponceau S solution (Sigma-Aldrich^®^) to check for transference efficacy, and blocked with a 3% bovine serum albumin (BSA) in TBS-T (Tris-Buffered Saline with Tween-20 containing in mM: Tris base 20; NaCl 137 and 0.1% Tween-20) during 1 h at RT to avoid non-specific binding. Membranes were incubated with the primary antibodies overnight at 4°C, and then with the HRP-conjugated secondary antibodies (1:10000, Santa Cruz Biotechnology, Dallas, TX, USA) for 1 h at RT, all diluted in 3% BSA solution in TBS-T. The primary antibodies used were rabbit polyclonal antibody anti-GFAP (1:5000, Sigma, St. Louis, MO, USA), goat polyclonal antibody anti-Iba-1 (1:1000, Abcam, Cambridge, UK), and mouse monoclonal antibody anti-GAPDH (1:5000, Invitrogen, Carlsbad, California, USA). Chemiluminescent detection was performed with ECL Plus Western Blotting Detection Reagent (GE Healthcare, Buckinghamshire, UK) in the ChemiDoc™ XRS^+^ System from Bio-Rad. The integrated intensity of each band was calculated using computer-assisted densitometry analysis with Image-J 1.45 software (Bethesda Softworks, Bethesda, MD, United States) and normalized to the integrated intensity of the loading control (GAPDH). Images were prepared for printing in Image Lab software 5.2.1 (software available in ChemiDoc XRS^+^ system, Bio-Rad).

### Immunohistochemistry

After behavioral tests, three animals of each group were deeply anesthetized with ketamine/xylazine mixture (120 mg/kg/16 mg/kg) at a volume of 1 ml/kg. After reaching a deep anesthesia state, the animals were perfused transcardially with approximately 200 ml of warmed (37°C) 0.9% saline solution, to clear the blood from the circulatory system, followed by approximately 500 ml of 4% paraformaldehyde (PFA) in phosphate buffer (PBS, 140 mM NaCl, 3 mM KCl, 20 mM Na_2_HPO_4_, 1.5 mM KH_2_PO_4_, pH 7.4) (Gage et al., 2012). Animals were then decapitated, and their brains were carefully removed and maintained for post-fixation in the same fixative solution at 4°C overnight. After that, brains were washed twice with PBS, and then cryoprotected at 4°C by immersion in increasing concentrations of sucrose (15% and 30%). Subsequently, brains were gelatin-embedded (7.5% gelatin in 15% sucrose) and then sectioned at a thickness of 12 µm on a cryostat (LEICA CM 3050S, Wetzlar, Germany), by the Histology and Comparative Pathology Laboratory of iMM. Only the coronal sections located at the level of hippocampus (around −2.92 mm and −5.04 mm from Bregma) were collected, mounted on SuperFrost^®^ Plus slides (Menzel-Glaser, Braunschweig, Germany) and stored at −20°C for further use.

For the immunohistochemical analyses, slices were placed in PBS for 10 min at 37°C to remove the gelatin from brain tissue. Then, each slice was surrounded with DAKO pen (Dako, Glostrup, Denmark) to protect staining areas from drying out and from mixing with each other. After an incubation in glycine (0.1 M) for 10 min to remove the small toxic aldehydes originated from PFA degradation, sections were subsequently treated with 0.1% Triton X-100 in PBS (10 min) for membrane permeabilization, washed twice (10 min each time) with PBS in the presence of 0.1% Tween-20 (PBS-T) and then blocked with a blocking solution (10% Fetal Bovine Serum (FBS), 6/10% BSA in PBS-T) for 30 min at RT. Next, slices were incubated with the primary antibodies overnight at 4°C, and with the fluorophores coupled-secondary antibodies for 2 h at RT in a humidified dark chamber. The nuclei were stained with Hoechst 33342 (20 μg/ml, Invitrogen) for 10 min at RT. The slices were mounted in Mowiol (Sigma). The primary antibodies used were mouse monoclonal antibody anti-GFAP (1:1000, Millipore, Burlington, Massachusetts, USA), and goat polyclonal antibody anti-Iba1 (1:1000, Abcam). The secondary antibodies were donkey anti-mouse-Alexa Fluor 568, and donkey anti-goat-Alexa Fluor 488 (1:500, Invitrogen). Images were acquired on an inverted wide field fluorescence microscope (Zeiss Axiovert 200, Zeiss, Oberkochen, Germany), using a monochrome digital camera (AxioCamMR3, Zeiss), with a 40× objective (Zeiss). The software AxioVision 4.7.1 (Carl Zeiss Imaging Systems) was used for image acquisition. Immunofluorescence images were acquired in two areas of the hippocampus: cornu ammonis 1 (CA1) and dentate gyrus (DG), of both hemispheres of each animal.

### Freshly Prepared Hippocampal Slices and Drug Treatment

Male C57BL/6J mice (8–11 weeks old), purchased to Charles River Laboratories (Lyon, France), were housed in groups of 4/5 per cage and maintained under controlled conditions (21 ± 1°C; 55 ± 10% humidity; 12:12 h light/dark cycle). Animals were deeply anesthetized with isoflurane (Esteve, Barcelona, Spain) and decapitated to quickly remove their brains. Then, brains were placed in ice-cold artificial cerebral-spinal fluid solution (aCSF; 124 mM NaCl, 3 mM KCl, 1.2 mM NaH_2_PO_4_, 25 mM NaHCO_3_, 2 mM CaCl_2_, 1 mM MgSO_4_, and 10 mM glucose, pH 7.4), continuously oxygenated (O_2_/CO_2_: 95%/5%) to dissect both hippocampi. Acute hippocampal slices were cut, with a thickness of 400 µm, perpendicularly to the long axis of the hippocampus using a McIlwain tissue chopper, and placed in a resting chamber filled with continuously oxygenated aCSF at RT for 1 h to guarantee the functional and energetic recovery. Slices were incubated for 3 h at RT with continuously oxygenated aCSF with 50 nM KTP-NH_2_ in the presence or absence of 200 nM Aβ_olig_.

### *Ex Vivo* Electrophysiological Recordings

Long-term potentiation (LTP) induction and quantification were performed as described previously (Diógenes et al., 2011). Briefly, hippocampal slices were transferred to a recording chamber continuously superfused with oxygenated aCSF at 32°C (flow rate of 3 ml/min). Stimulation pulses were delivered every 20 s using a bipolar concentric wire electrode placed on Shaffer collateral/commissural fibers in stratum radiatum, and field-excitatory post-synaptic potentials (fEPSPs) were recorded extracellularly through a microelectrode filled with aCSF (2–6 MΩ) placed in the stratum radiatum of the CA1 area. The intensity of stimulation was initially adjusted to obtain a fEPSP slope with a minimal spike contamination and of around 50% of the maximal slope. Recordings were obtained with an Axoclamp 2B amplifier (Axon Instruments, Foster City, CA, United States), digitized, and continuously stored on a personal computer with the WinLTP software (Anderson and Collingridge, 2001). Individual responses were monitored, averages of six consecutive responses were obtained, and the slope of the initial phase of the fEPSP was quantified. After fEPSPs stabilization, LTP was induced through a θ-burst protocol (3 trains of 100 Hz, 3 stimuli, separated by 200 ms). The θ-burst induced LTP pattern of stimulation is considered closer to what physiologically occurs in hippocampi during episodes of learning and memory in living animals (Albensi et al., 2007). We used a mild LTP protocol since this proved to be sensitive to synaptic plasticity changes along with aging (Diógenes et al., 2011), and allows assessing both further improvements or impairments on LTP magnitude. LTP magnitude was quantified as the percentage of change in the average slope of fEPSPs taken between 50 to 60 min after LTP induction in relation to the average slope of fEPSPs measured during the 10 min before θ-burst induced LTP (baseline).

### Primary Neuronal Cultures and Drug Treatment

Primary neuronal cultures were obtained from fetuses of 18/19-days pregnant Sprague-Dawley females, as routinely in the lab (Jerónimo-Santos et al., 2015). Briefly, animals were deeply anaesthetised with isoflurane and decapitated. The fetuses were collected and placed in cold Ca^2+^- and Mg^2+^-free Hank’s Balanced Salt Solution (HBSS) (Gibco, Paisley, UK). The brains were dissected, the cerebral cortices were isolated, and the meninges were removed. Then, the tissue was mechanically fragmented, followed by enzymatic digestion with 0.025% (w/v) of trypsin solution in HBSS for 15 min at 37°C. To neutralize the action of trypsin, HBSS supplemented with FBS 20% (w/v) and cellular suspension was centrifuged at 190*g*. The supernatant was discarded, and the cells were resuspended in the same solution by pipette aspiration in order to dissociate cells. This process was repeated three times. After washing, cells were resuspended in supplemented Neurobasal medium (0.5 mM l-glutamine, 25 mM glutamic acid, 2% B-27, and 25 U/ml penicillin/streptomycin). Then, cell suspension was filtered (BD Falcon Cell Strainer 70 mM, Thermo Fisher Scientific, Waltham, MA, United States) to obtain single cells, and cell density was determined by counting cells in a 0.4% trypan blue solution using a hemocytometer. Cells were plated at 1 × 10^−5^ cells/well on 12-wells flat-bottom cell plates covered with glass coverslips (Corning^®^ Costar^®^ TC-treated, Sigma), and maintained at 37°C in a humidified atmosphere of 95/5% O_2_/CO_2_, for 14 days. The coverslips were previously sterilized under UV light, coated overnight with 10 mg/ml of poly-d-lysine (Sigma, St. Louis, MO, United States) and washed with sterile H_2_O. After 13 days *in vitro* (DIV13), cells were incubated with 50 nM KTP-NH_2_ in the presence or absence of 25 µM Aβ_25–35_, during 24 h.

### Immunocytochemistry

In order to evaluate the neuroprotective effect of drug treatment, spine density was counted as previously done in our lab (Tanqueiro et al., 2018). Briefly, primary neuronal cultures at DIV14 were fixed in 4% PFA in PBS (pH 7.4) for 15 min at RT, after being washed with PBS twice. Using a blocking solution (3% (w/v) BSA) (Sigma-Aldrich) in PBS with 0.1% (v/v) Triton X-100, cells were incubated for 1 h at RT. To specifically detect neurons, cells were incubated with mouse microtubule-associated protein 2 (MAP2) primary antibody (1:200 in blocking solution) (Millipore), overnight at 4°C in a wet-chamber. After this, cells were washed with PBS twice and then co-incubated in blocking solution with goat anti-mouse-Alexa Fluor 568 secondary antibody (1:200, Invitrogen), Alexa Fluor 488 Phalloidin (1:40) (Invitrogen), which recognizes filamentous actin (F-actin), and Hoechst 33342 (6 µg/ml) for nuclear staining, for 1 h at RT inside a dark-wet chamber. After being washed with PBS twice, coverslips were mounted in Mowiol mounting solution. Using a confocal laser point-scanning microscope LSM 880 with Airyscan (Carl Zeiss MicroImaging, Thornwood, NY, United States), the observed conjugation between MAP2 (568, red) and F-actin (488, green) allowed the clear identification of dendritic protrusions, since F-actin has an important role in the constitution of the cytoskeleton of dendritic spines (Koskinen and Hotulainen, 2014). Spine density was assessed as the number of protrusions, counted per 10 µm of the parent dendrite, as previously reported (Alonso et al., 2004; Ji et al., 2010) with a distance of 25 µm from the cell body. Independent averages were computed with counts of 3 different dendrites of each neuron, 6 neurons per condition, with each primary neuronal culture being considered an independent experiment.

### Statistical Analysis

Data are expressed as mean ± standard error of the mean (mean ± SEM), where *n* is the number of independent experiments. Results of each animal per group, and results acquired from different hippocampal slices of different animals were considered independent experiments. The data normality was confirmed using the Shapiro-Wilk test. Results obtained using behavioral paradigms, OFT, YM-SAT, NORT (object recognition index and object discrimination index), through electrophysiological recordings (LTP magnitude), and using immunocytochemistry (number of dendritic protrusions) were analyzed using two-way ANOVA, followed by the Tukey’s multiple comparisons test. In addition, significant differences between means of NORT object preference index within each phase were evaluated through unpaired two-tailed Student’s *t*-test. Results obtained by Western blot (WB) were analyzed using two-way ANOVA. Values of p < 0.05 were considered to represent statistically significant differences. All statistical analyses were conducted using the Prism Software (GraphPad Prism^®^, version 8.0.2, California, USA).

## Results

### KTP-NH_2_ Treatment Prevented Episodic Long-Term and Spatial Working Memory Dysfunction Induced by Aβ

To evaluate the influence of KTP-NH_2_ in an animal model of AD (Canas et al., 2009; Zhang et al., 2015), KTP-NH_2_ (32.3 mg/kg) or saline solution (vehicle) was i.p. administered for 18 days to rats that had received a 5 µl i.c.v injection of Aβ_1–42_ (2.25 mg/ml) or water (vehicle) (**Figure 1A**). As such, the experimental groups are the following: control group (vehicles); KTP-NH_2_ group (vehicle + KTP-NH_2_ treatment); Aβ group (Aβ and vehicle treatment); and Aβ + KTP-NH_2_ group (Aβ and KTP-NH_2_ treatment) (**Figure 1B**).

**Figure 2:**
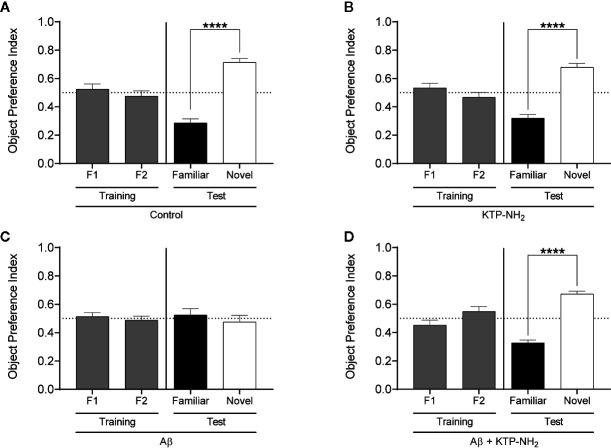
The KTP-NH_2_ treatment mitigates Aβ-induced impairments in long-term (24 h) episodic memory. Long-term episodic memory was evaluated through the Novel Object Recognition test (NORT). After 3 arena habituation sessions, the training phase consisted of one session with two identical objects (F1 and F2) placed inside the arena. The test phase occurred on the following day (24 h later), where one of the familiar objects (Familiar) was replaced by a novel object (Novel). During both phases, animals freely explore the objects for 5 min and the results are given by the time spent exploring any one of the two objects allows to calculate the Object Preference Index, i.e., F1 or F2 / (F1 + F2) and Familiar or Novel / (Familiar + Novel). A preference index above 0.5 indicates a preference of that specific object, whereas equal to 0.5 represents no preference. Statistical analysis using unpaired two-tailed Student’s t-test showed no statistical differences in preference between objects during the training phase (as expected), and revealed a significant preference for the novel objects during the test phase for control **(A)**, KTP-NH_2_ **(B)**, and Aβ + KTP-NH_2_ **(D)** groups (n = 6–7), which indicates that memory is conserved within these animals. For the Aβ group **(C)**, data showed that there was no preference between familiar and novel objects (n = 6). ****p ≤ 0.0001. Data are represented as mean ± SEM.

Episodic long-term memory, considered the predominant cognitive deficit in AD, was evaluated through the NORT with a retention time of 24 h (see Methods). As expected, in the training phase (**Figure 2**, gray bars), the four groups of animals explored approximately the same amount of time the two identical objects. In the test day (**Figure 2**, black and white bars), animals from the Aβ group did not react to novelty (p > 0.05, unpaired two-tailed Student’s *t*-test to compare % of time spent with familiar *vs*. novel object; n = 6; **Figure 2C**), whereas the control group did (p ≤ 0.0001, unpaired Student’s *t*-test to compare % of time spent with familiar *vs*. novel object; n = 7; **Figure 2A**). Remarkably, the Aβ induced-impairment in the NORT was totally recovered in the animals that had been treated with KTP-NH_2_ (**Figure 2D**). These treated animals showed a clear preference for exploring the novel object more than the familiar one (p ≤ 0.0001, unpaired Student’s *t*-test to compare % of time spent with familiar *vs*. novel object; n = 6), thus behaving like control animals. The KTP-NH_2_ group also behaved like a control group (p > 0.05, unpaired two-tailed Student’s *t*-test to compare % of time spent with familiar *vs*. novel object; n = 7; **Figure 2B**), indicating that *per se* KTP-NH_2_, is not a memory enhancer, though markedly ameliorating memory deficits elicited by Aβ.

**Figure 3:**
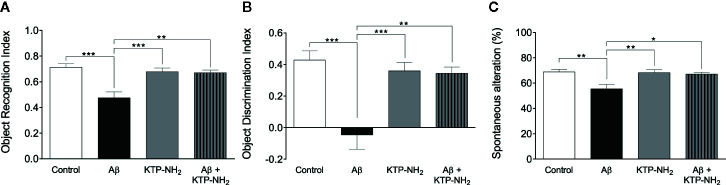
KTP-NH_2_ treatment mitigates Aβ-induced impairments in long-term episodic memory and spatial working memory. **(A)** Object recognition index was calculated from the data obtained in the test day of the Novel Object Recognition test (NORT), as [novel / (novel + familiar)], and it ranges from 0 to 1 (where 1 means only exploration of the novel object and zero means only exploration of the familiar object). **(B)** Object discrimination index was also calculated from the data obtained in the test day of NORT, as [(novel – familiar) / (novel + familiar)], and it ranges from −1 to 1, where zero means no discrimination between both objects. **(C)** The percentage of spontaneous alternation was calculated from data obtained during the Y-Maze Spontaneous Alternation test (YM-SAT), as actual alternations/maximum alternations x 100. For each statistical analysis, significant differences between the group of Aβ animals (n = 6) and all the other groups (n = 6–7) were assessed using two-way ANOVA followed by Tukey’s multiple comparisons test. *p < 0.05, **p ≤ 0.01, ***p ≤ 0.001. Data are represented as mean ± SEM.

To better compare performances among the four groups of animals, the time spent exploring each object in the test day was converted into the object recognition index [novel/(novel + familiar)], and the object discrimination index [(novel – familiar)/(novel + familiar)], which were calculated for each animal and averaged within each group (**Figures 3A, B**, respectively). The object recognition index ranges from 0 to 1, where obtained values around 0.5 reflect an absence of discrimination between the novel and the familiar objects. The discrimination index ranges from −1 to +1, and obtained values around 0 reflect lack of object discrimination. In both indexes, higher obtained values indicate higher memory performance. As depicted in **Figure 3**, the recognition index attained by the Aβ group was significantly different from the control group (Aβ: 0.48 ± 0.046 *vs.* control: 0.71 ± 0.028; p < 0.05, two-way ANOVA with Tukey’s multiple comparisons test; n = 6–7). Importantly, the index displayed by the Aβ + KTP-NH_2_ group, (0.67 ± 0.020, n = 6) was similar to the control group (p > 0.05, two-way ANOVA with Tukey’s multiple comparisons test; n = 6–7), and significantly different from Aβ group (p < 0.05, two-way ANOVA with Tukey’s multiple comparisons test; n = 6–7). Moreover, the KTP-NH_2_ treatment alone (KTP-NH_2_ group) did not affect the object recognition index (0.68 ± 0.028, n = 7) when compared to the control group (p > 0.05, two-way ANOVA with Tukey’s multiple comparisons test; n = 6–7). Thus, this analysis further confirms that KTP-NH_2_ treatment had beneficial effects on the performance of Aβ i.c.v. injected animals in long-term memory testing. Similar findings were obtained by calculated scores of the discrimination index (control: 0.43 ± 0.058; Aβ: −0.05 ± 0.091; KTP-NH_2_: 0.36 ± 0.053; and Aβ + KTP-NH_2_: 0.35 ± 0.039, two-way ANOVA with Tukey’s multiple comparisons test; n = 6–7).

To determine whether KTP-NH_2_ treatment could counteract the Aβ induced-impairments in spatial working memory, animals were subjected to the YM-SAT, and their performance was assessed through the percentage of spontaneous alternation as described in methods (**Figure 3B**). As expected, animals from Aβ group had a lower percentage of spontaneous alternation when compared with animals from the control group (Aβ: 55 ± 3.5 *vs.* control: 69 ± 2.0, p < 0.05, two-way ANOVA with Tukey’s multiple comparisons test; n = 6–7), confirming that Aβ i.c.v. injected animals had an impairment in spatial working memory. However, when Aβ i.c.v. injected animals were treated with KTP-NH_2_ (Aβ + KTP-NH_2_ group) the percentage of spontaneous alternation in the YM-SAT (67 ± 1.3, n = 6) was similar to the control group (p > 0.05, two-way ANOVA with Tukey’s multiple comparisons test; n = 6–7), and significantly different from that of the Aβ group (p < 0.05, two-way ANOVA with Tukey’s multiple comparisons test; n = 6–7), implying that KTP-NH_2_ treatment reduced the Aβ induced-deficits in YM-SAT. Moreover, KTP-NH_2_ alone had no significant effect, since the percentage of spontaneous alternation of animals from KTP-NH_2_ group (68 ± 2.4, n = 7) was not significantly different from the control group (p > 0.05, two-way ANOVA with Tukey’s multiple comparisons test).

**Figure 4:**
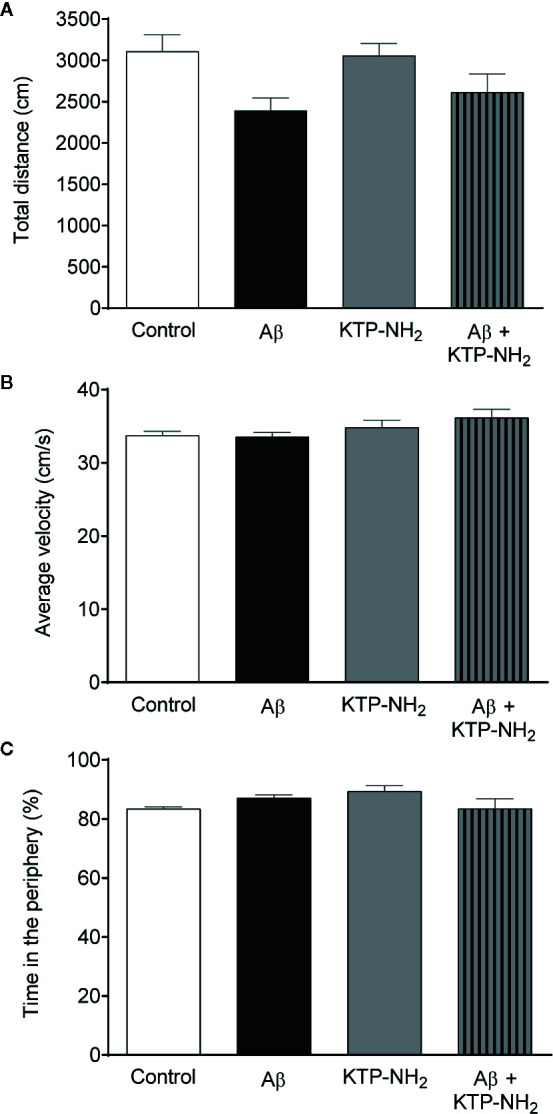
Neither Aβ nor KTP-NH_2_ treatment affected locomotor activity or anxiety-like behaviour. Results obtained during the Open-field test (OFT) performance allow the assessment of locomotor activity through the **(A)** total distance travelled (cm), and **(B)** average velocity (cm/s). **(C)** Anxiety-like behaviour was evaluated through the percentage of time spent in the periphery of the OFT. Statistical analysis using two-way ANOVA did not reveal significant differences (p < 0.05) between groups (n = 6-7). Data are represented as mean ± SEM.

Performance in the NORT and in the YM-SAT can be affected by changes in locomotion, by anxiety-like behavior, or by alterations in exploratory drive. To control for these parameters, animals were assessed in the OFT. No statistically significant differences (**Figure 4**; p > 0.05, two-way ANOVA; n = 6–7) were found between any of the experimental groups when analyzing the total distance travelled (**Figure 4A**), or the average velocity (**Figure 4B**). In addition, the total number of entries in the YM-SAT arms was also analyzed (control: 26 ± 1.6; Aβ: 23 ± 1.5; KTP-NH_2_: 25 ± 2.0; Aβ + KTP-NH_2_: 21 ± 1.5), and no significant differences were found between any of the groups (p > 0.05, two-way ANOVA; n = 6–7). Together, these results show that neither Aβ, nor KTP-NH_2_, alone or in combination, caused appreciable effects in locomotor activity that could mask performance in the NORT or YM-SAT. Similarly, no statistically significant differences (p > 0.05, two-way ANOVA, n = 6–7) were found between groups for the percentage of time spent in the periphery of the OFT arena (**Figure 4C**), which also indicates an absence of appreciable changes in anxiety-like behaviour.

### Hippocampal Gliosis Immediately After Testing Was Not Different Among the Groups

**Figure 5:**
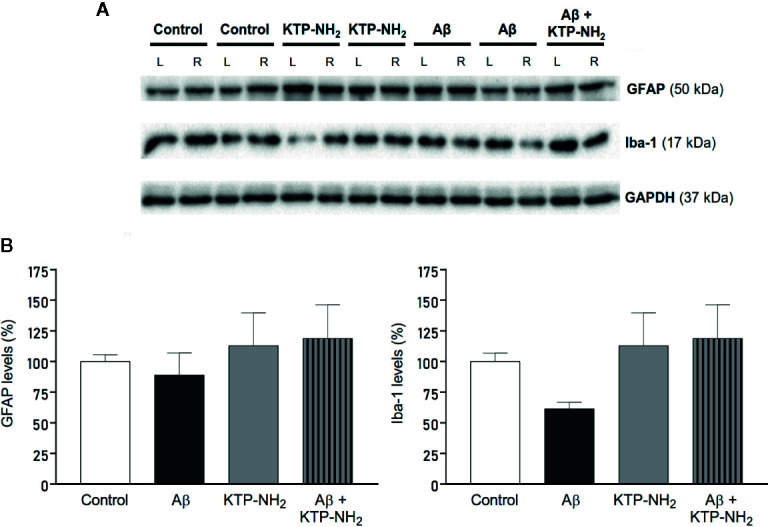
Neither Aβ nor KTP-NH_2_ affected hippocampal gliosis assessed by immunoblotting. Protein levels were assessed by western blotting using tissue homogenates from each isolated hippocampus of 13 animals, 3-4 for each experimental group (Control, KTP-NH_2_, Aβ, and Aβ + KTP-NH_2_). **(A)** Representative immunoblots of GFAP (50 kDa), Iba-1 (17 kDa) and GAPDH (37 kDa). GAPDH was used as a loading control. **(B)** Densitometry analysis of the right (R) (side of i.c.v injection) hippocampus, performed for all groups with ImageJ software. No statistically significant differences were found between animal groups regarding GFAP or Iba-1 immunoreactivity and (p > 0.05, two-way ANOVA, n = 3-4). Data are represented as mean ± SEM.

**Figure 6:**
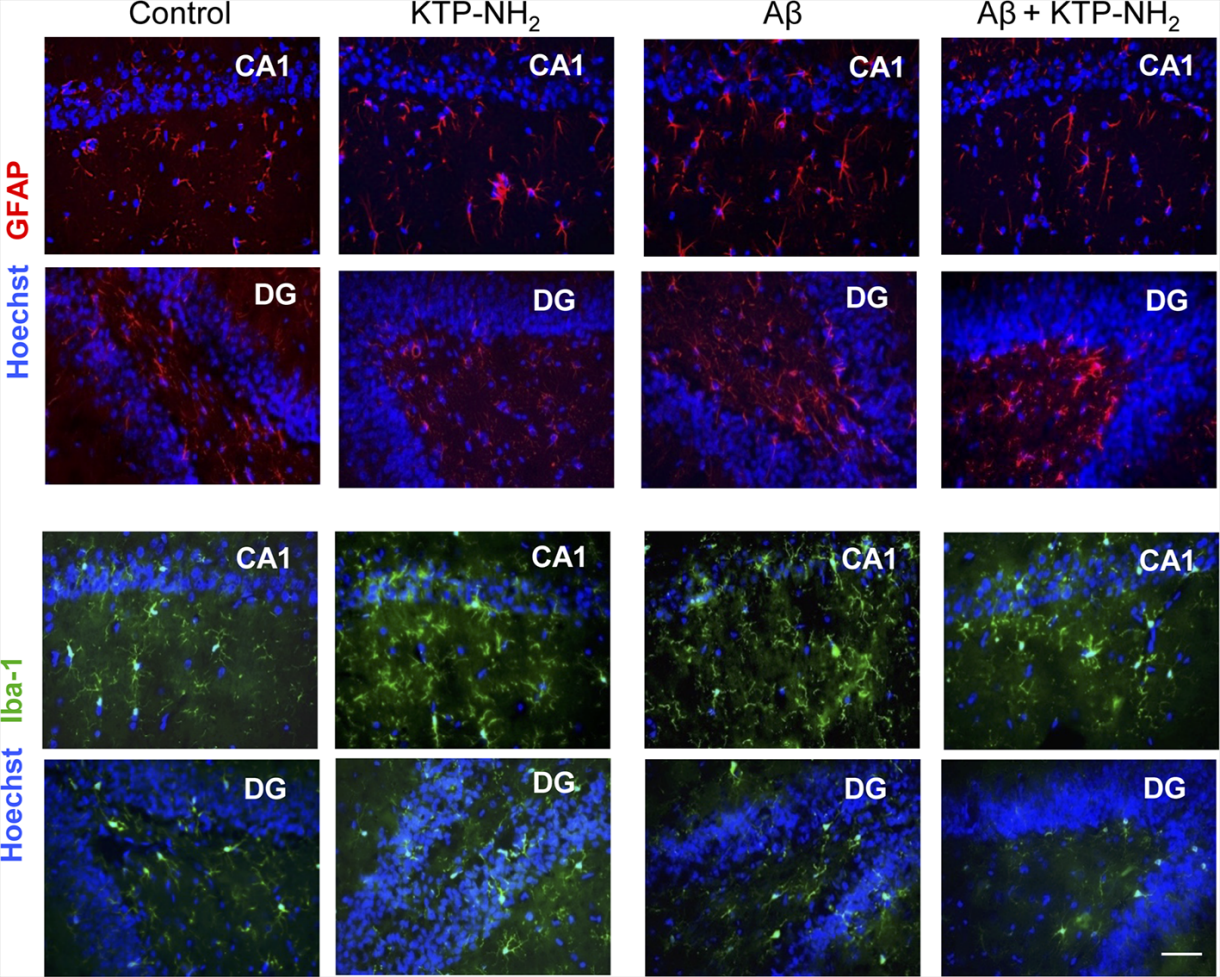
Neither Aβ nor KTP-NH_2_ affected hippocampal gliosis assessed by immunohistochemistry labeling. At day 21, thirteen deeply anesthetized animals were transcardially perfused with ice-cold saline and 4% PFA solutions. After decapitation, brains were removed and gelatin-embedded to be sectioned on a cryostat. Coronal sections located at hippocampus level were collected and mounted for labelling with GFAP to stain astrocytes (red) and Iba-1 to stain microglia (green). Nuclei were stained with Hoechst (blue). Images of both CA1 and DG hippocampal areas were acquired on an inverted wide-field fluorescence microscope (Zeiss Axiovert 200), with a 40x objective. The figure includes representative images of each experimental group (Control, KTP-NH_2_, Aβ, and Aβ + KTP-NH_2_). There was no difference in GFAP or Iba-1 staining between conditions. Scale bar, 50 µm.

To assess if Aβ could lead to an increase in the proliferation of astrocytes and microglia at the time of testing, which could suggest gliosis and thus inflammation, WB assays were performed with protein extracts obtained from hippocampal homogenates prepared upon animal sacrifice after behavioral testing. GFAP and Iba-1 were used as markers for astrocytes and microglia, respectively (**Figure 5**). Densitometry analyses of the WB revealed no significant changes (n=3–4; p > 0.05, two-way ANOVA) in samples from Aβ injected animals when compared with control, and in response to KTP-NH_2_ treatment, thus indicating an absence of proliferation of astrocytes and microglia cells. Nevertheless, since WB assay did not provide information about morphological changes of astrocytes and microglia, immunohistochemistry analysis of glial cell morphology was performed to further address this issue. As shown in **Figure 6**, regardless of the hippocampal area analyzed (CA1 or DG), there was no evidence of astrogliosis (evaluated by GFAP immunoreactivity) or microgliosis (evaluated by Iba-1 immunoreactivity), either in control or Aβ animals, treated or untreated with KTP-NH_2_, since no morphologic differences in glial cells were noticeable.

### KTP-NH_2_ Prevented the Impairment in θ-Burst-Induced LTP Magnitude Caused by Aβ

**Figure 7:**
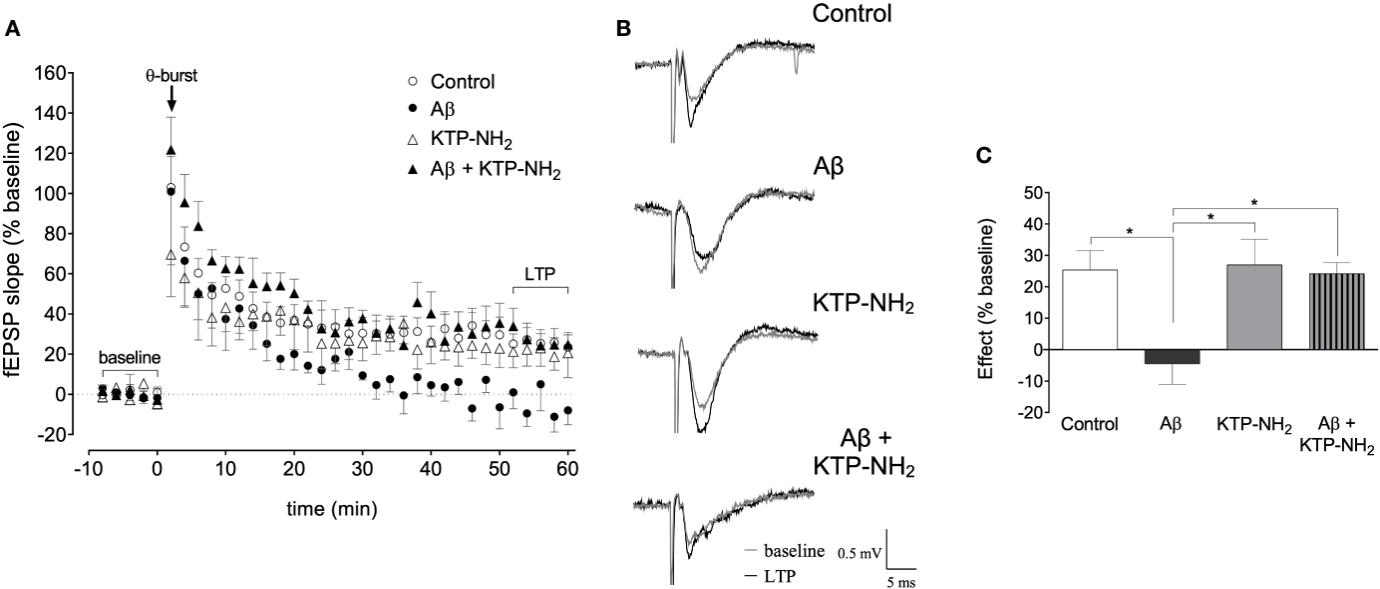
Incubation with KTP-NH_2_ reduces the impact of Aβ on LTP magnitude upon θ-burst-induced LTP. **(A)** The averaged time courses changes in field excitatory post-synaptic potential (fEPSP) slope (% baseline) induced by a θ-burst stimulation in animal hippocampal slices with a pre-exposure of 3 h to aCSF solution (control, n = 8) with 200 nM Aβ_olig_ (Aβ, n = 5), 50 nM KTP-NH_2_ (KTP-NH_2_, n = 4), or 200 nM Aβ_olig_ and 50 nM KTP-NH_2_ (Aβ + KTP-NH_2_, n = 5). **(B)** Tracings from representative experiments. For each condition, fEPSP tracings recorded at baseline (baseline, grey line) and after θ-burst-induced LTP (LTP, black line) from the same slice are showing overlaid. **(C)** Histogram depicting the effect on LTP magnitude (% average changes in fEPSP slope at 50-60 min normalized to the defined baseline of 10 min immediately before θ-burst stimulation) regarding each group under study (control, Aβ, KTP-NH_2_, Aβ + KTP-NH_2_). Statistical analysis using two-way ANOVA followed by Tukey’s multiple comparisons test indicates a significant difference between the Aβ condition (n = 5), and all the other groups (n = 4-8). *p < 0.05. Data are represented as mean ± SEM.

To address the synaptic mechanisms involved in the ability of KTP-NH_2_ to mitigate memory impairment in animals injected with Aβ, we assessed the effect of KTP-NH_2_ upon θ-burst-induced LTP in hippocampal slices that had been incubated with 200 nM Aβ_olig_ (**Figures 7A, B**), a known to be toxic conformational arrangement of Aβ (Giuffrida et al., 2009).

LTP magnitude (**Figure 7C**), recorded in slices pre-incubated with 200 nM Aβ_olig_ for 3 h (Aβ) was significantly decreased when compared to that attained in control slices incubated for a similar amount of time in the absence of Aβ (control: 25.4 ± 6.15 *vs.* Aβ: −4.52 ± 6.53, p < 0.05, two-way ANOVA with Tukey’s multiple comparisons test; n = 5–8). Remarkable, in slices co-incubated with 200 nM Aβ_olig_ and 50 nM KTP-NH_2_ (Aβ + KTP-NH_2_), LTP magnitude (24.2 ± 3.50, n = 5) attained values similar to those in control slices (p > 0.05, two-way ANOVA with Tukey’s multiple comparisons test; n = 5–8). Moreover, LTP magnitude in slices pre-incubated with only 50 nM KTP-NH_2_ (KTP-NH_2_) was similar (27.0 ± 8.10, n = 4) to that in control slices (p > 0.05, two-way ANOVA with Tukey’s multiple comparisons test; n = 5–8). Altogether, these results clearly indicate that KTP-NH_2_ prevents Aβ-induced impairment of LTP without by itself affecting LTP.

### KTP-NH_2_ Prevented the Decrease in Spine Density Caused by Aβ Action on Cultured Cortical Neurons

**Figure 8:**
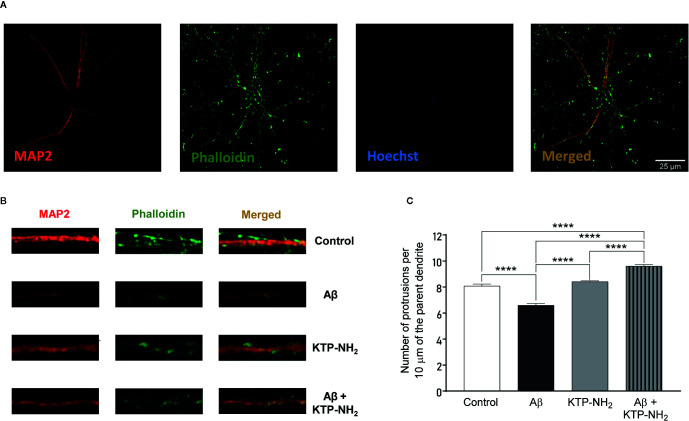
Incubation with KTP-NH_2_ mitigates Aβ incubation-induced impairments in spine. **(A)** Representative image of an untreated neuron (control) obtained from primary neuronal cultures. Primary neuronal cultures (DIV13) were incubated with 25 µM Aβ_25-35_ and/or with 50 nM KTP-NH_2_ for 24 h. MAP2 (red) specifically labels neurons, while phalloidin (green) labels F-actin. The merge between both labels (yellow) allows the identification of dendritic protrusions. To evaluate spine density, the number of protrusions *per* 10 µm of the parent dendrite with a distance of 25 µm from the cell body were counted (3 parent dendrites *per* neuron, 6 neurons *per* condition). **(B)** Treatment effects on synaptic density (10 µm). **(C)** Histogram depicting spine density as the number of protrusions from each condition. Statistical analysis using two-way ANOVA followed by Tukey’s multiple comparisons test indicates a significant difference between conditions (n = 5). While 25 µM Aβ_25-35_ treatment (Aβ) diminished the number of protrusions in cortical neurons, 50 nM KTP-NH_2_ co-treatment (Aβ + KTP-NH_2_) restored spine density. In addition, 50 nM KTP-NH_2_ treatment (KTP-NH_2_) did not, by itself, impact spine density. ****p ≤ 0.0001. Data are represented as mean ± SEM.

To further investigate the synaptic correlates of the protective action of KTP-NH_2_ against Aβ-induced synaptic impairment, we evaluated the effect of KTP-NH_2_ on spine density of cultured cortical neurons. The conjugation between MAP2 (red) and Phalloidin (green) labels, allowed the identification of dendritic protrusions (yellow) (**Figure 8A**), as a morphological readout of the treatment modulation of the number of dendritic spines. The representative segments of 10 µm of counted dendrites for each analysed condition are illustrated in **Figure 8B**.

In accordance with previous studies (Tanqueiro et al., 2018), cultured cortical neurons exposed to Aβ_25–35_ for 24 h, showed a significant decrease in the number of dendritic protrusions when compared to the control condition (Aβ: 6.58 ± 0.153 *vs.* control: 8.07 ± 0.153; p < 0.05, two-way ANOVA with Tukey’s multiple comparisons test; n = 5) (**Figure 8C**). Remarkably, the Aβ-induced impairment in the number of dendritic protrusions was absent when neurons were co-treated with 50 nM KTP-NH_2_ (Aβ + KTP-NH_2_: 9.60 ± 0.134 *vs.* control: 8.07 ± 0.153; p < 0.05, two-way ANOVA with Tukey’s multiple comparisons test; n = 5). KTP-NH_2_ alone did not significantly alter dendritic protrusions when compared to the control condition (KTP-NH_2_: 8.41 ± 0.071 *vs.* control: 8.07 ± 0.153; p > 0.05, two-way ANOVA with Tukey’s multiple comparisons test; n = 5).

## Discussion

A main finding in the present work is that systemically applied KTP-NH_2_ prevents memory dysfunction induced by Aβ. At the synaptic level we could demonstrate that KTP-NH_2_ prevents synaptic plasticity and spine density impairments caused by Aβ, suggesting that KTP-NH_2_ mitigates memory deficits by protecting hippocampal synapses against Aβ-induced toxicity.

Since the hippocampus is required for performance in the NORT (Goulart et al., 2010), this test has been considered a useful tool for basic and preclinical research in the context of AD (Rajagopal et al., 2014). In addition, the sustained strengthening of synaptic connections that characterizes hippocampal long-term potentiation (LTP) is taken as a synaptic correlate of the basic mechanisms involved in memory and learning processes (Bliss and Collingridge, 1993). Accordingly, abnormal performance in memory tasks, especially hippocampal-dependent ones (Figurov et al., 1996; Korte et al., 1998; Pang and Lu, 2004), are associated with impairments in LTP (Morris et al., 1986). Our data showing that KTP-NH_2_ prevented the Aβ-induced impairment of hippocampal LTP, strongly suggest that the reestablishment of synaptic plasticity is one of the mechanisms underlying the cognitive actions of KTP-NH_2_. Furthermore, at a molecular level, neuronal spine density has been associated with synaptic reinforcement during LTP (Toni et al., 1999; Luscher and Malenka, 2012). In fact, spine density is known to be reduced in cultured cortical neurons incubated with Aβ peptide (Tanqueiro et al., 2018). Our results show that KTP-NH_2_ restores Aβ-induced decrease in spine density, further explaining its neuroprotective actions upon synaptic plasticity. These results pave the way for further studies on the mechanism of action of KTP-NH_2_ addressing if it reestablishes spine density *in vivo*.

While the presence of episodic memory impairments mimics the clinical hallmarks detected in AD patients in the early stages of the disease (Alescio-Lautier et al., 2007; Gold and Budson, 2008; Huntley and Howard, 2010), motor dysfunctions are better noticed in moderate to severe stages of AD (Zidan et al., 2012). In the present work, we could detect episodic memory dysfunctions through the YM-SAT and NORT, without signs of clear motor dysfunctions, judged by OFT performance. This indicates that the sporadic AD model used mostly mimics the earlier stages of AD. It is known that loss of synapses, mainly in the hippocampus, is one of the earliest consequences of Aβ toxicity (Canas et al., 2009), and that this loss correlates with the initial memory impairments in AD patients (Coleman et al., 2004). Furthermore, compared to the transgenic models of AD available, the use of this sporadic AD model, where the Aβ injection is responsible for the induced-pathophysiological traits of AD, allows us to have a direct understanding of the effect of KTP-NH_2_ administration on the Aβ-induced dysfunction. Our findings support that systemic administration of KTP-NH_2_ is able to prevent memory loss as well as synaptic plasticity dysfunctions induced by Aβ, highlight the therapeutic potential of BBB permeable KTP analogues in early stages of AD. This may gain particular relevance since the few therapeutic tools so far available for early disease stages are mostly symptomatic to increase cholinergic function. As we show, the KTP derivative is able to enhance synaptic plasticity and, importantly, to prevent retraction of dendritic spines in cultured neurons, thus to prevent synaptic atrophy, a clear sign of neuroprotective ability.

Furthermore, KTP biosynthesis occurs in the nerve terminals (Ueda et al., 1986), which is especially relevant in AD pathophysiology, where the loss of cortical mass evolves fast during the early stages of the disease (Sabuncu, 2011). This cellular death implies less KTP production capability, resulting in a decrease of KTP concentration in the CSF of AD patients (Santos et al., 2013). Accordingly, the increase of KTP levels in the brain emerged as a possible therapeutic tool for AD treatment. However, KTP has pharmacokinetic hindrances, such as its limited capacity to cross the BBB and its high susceptibility to clearance mechanisms, which hampered its clinical use (Serrano et al., 2015). Our work shows that it is possible to circumvent this problem by using a BBB permeant KTP analogue, KTP-NH_2_. The introduced structural change increased the peptide global net charge, increasing its capacity to interact with biological membranes, most notably giving it the ability to cross the BBB (Ribeiro et al., 2011a).

Besides having an acute and chronic well-characterized analgesic-profile when systemically administrated (Ribeiro et al., 2011a; Ribeiro et al., 2011b), this small and cost-effective molecule also showed a remarkable anti-inflammatory effect. Accordingly, independent studies developed by our lab showed that KTP-NH_2_ impacts the glucocorticoid system, which might account for the molecular link between analgesia, anti-inflammation, and neuroprotection (Perazzo et al., 2017). Neuroinflammation caused by Aβ has been commonly linked to the presence of gliosis and, in some studies, to neuronal damage. In the present work, gliosis was evaluated on day 21 after Aβ i.c.v. injection by both immunohistochemistry and Western blotting and, in agreement with previous data (Canas et al., 2009), no significant signs of astrogliosis nor microgliosis were detected in the hippocampus. However, it is important to highlight that the peak of the inflammatory response after Aβ injection is one week after injection (McLarnon, 2014). So, we cannot discard initial Aβ-induced neuroinflammation nor an influence of KTP-NH_2_ that may have occurred before memory assessment. Nevertheless, our results show that at least immediately after memory testing, there were no signs of gliosis.

In summary, we clearly show that, in a sporadic AD model, systemic administration of KTP-NH_2_ protects spatial working- and episodic memory, without affecting motor activity or inducing anxiety-like behavior. Moreover, KTP-NH_2_ prevented Aβ-induced deficits in LTP magnitude and in spine density. We, therefore, highlight a neuroprotective action of KTP-NH_2_ against the early stages of AD pathophysiology. While these results are promising as far as pharmacodynamics is concerned, they clearly push toward the need of further studies on the pharmacokinetics of KTP-NH_2_ after its systemic administration through different routes, as well as to the need of other studies directed toward other hallmarks of AD. Indeed, in light of the complex pathophysiology of AD, it is important to know whether KTP-NH_2_ has a greater potential as a standalone treatment, or rather as part of a broader multidrug treatment regimen.

## Data Availability Statement

The raw data supporting the conclusions of this article will be made available by the authors, without undue reservation, to any qualified researcher.

## Ethics Statement

The animal study was reviewed and approved by Ethical Committee for Animal Research of Instituto de Medicina Molecular João Lobo Antunes (iMM), Faculty of Medicine, University of Lisbon, and the Portuguese Competent Authority for Animal Welfare (DGAV) in Portugal.

## Author Contributions

RFB and MLFM contributed equally to this work. Both performed the experiments, interpreted the results, and wrote the initial draft of the manuscript. RFB performed electrophysiological recordings and immunocytochemistry experiments as well as all statistical analyses. MLFM performed the i.c.v. injection of Aβ together with SX and JEC, the animal behavioral studies with LS under the supervision of JEC, and Western blots and immunohistochemistry procedures under the guidance of CAV. RFB and JF-G were responsible by the neuronal cell cultures. TC-C and CA-B contributed equally to immunocytochemistry analysis. HVM was responsible for preparing oligomeric species of Aβ with the supervision of TFO. CAV contributed to the interpretation of Western blot and immunohistochemistry experiments. VN contributed to the interpretation of the results. MH and EB were responsible for the synthetize of the KTP-NH_2_ peptide. MJD was responsible for the concept and design of the study regarding electrophysiological and immunocytochemistry experiments, contributed to the interpretation of the results. MARBC and AMS were responsible for the study design, interpreted the results and supervised the work. All authors contributed to the article and approved the submitted version.

## Funding

This study was supported by Santa Casa da Misericórdia de Lisboa (MB37-2017) and Fundação para a Ciência e Tecnologia (FCT): PTDC/NEU-OSD/5644/2014, and PTDC/BIA-VIR/29495/2017. RFB was supported by FCT (PD/BD/114337/2016). JF-G was supported by FCT (PD/BD/114441/2016). JEC was supported by FCT (SFRH/BPD/87647/2012).

## Conflict of Interest

The authors declare that the research was conducted in the absence of any commercial or financial relationships that could be construed as a potential conflict of interest.
